# Peptide Fraction from *Naja mandalayensis* Snake Venom Showed Neuroprotection Against Oxidative Stress in Hippocampal mHippoE-18 Cells but Not in Neuronal PC12 Cells

**DOI:** 10.3390/antiox14030277

**Published:** 2025-02-26

**Authors:** Brenda R. Silva, Lais C. Mendes, Marcela B. Echeverry, Maria Aparecida Juliano, Emidio Beraldo-Neto, Carlos Alberto-Silva

**Affiliations:** 1 Experimental Morphophysiology Laboratory, Natural and Humanities Sciences Center (CCNH), Universidade Federal do ABC (UFABC), São Bernardo do Campo 09606-070, SP, Brazil; brenda.rufino@ufabc.edu.br; 2 Biochemistry Laboratory, Butantan Institute, São Paulo 05503-900, SP, Brazil; lais.mendes.esib@esib.butantan.gov.br (L.C.M.); emidio.beraldo@butantan.gov.br (E.B.-N.); 3 Center for Mathematics, Computation and Cognition (CMCC), Universidade Federal do ABC UFABC, São Bernardo do Campo 09606-070, SP, Brazil; marcela.echeverry@ufabc.edu.br; 4 Departament of Biophysical, Escola Paulista de Medicina, Instituto de Ciências Ambientais, Químicas e Farmacêuticas, Universidade Federal de São Paulo, São Paulo 04023-062, SP, Brazil; ma.juliano@unifesp.br

**Keywords:** *Naja mandalayensis*, neuroprotective, bioactive peptide, reactive oxygen species, label-free analysis

## Abstract

Functional characterization of peptide fraction (PF) from snake venom has provided novel opportunities to investigate possible neuroprotective compounds relevant to pharmaceuticals. This study was performed to investigate the PF-mediated neuroprotection obtained from *Naja mandalayensis* snake venom, a member of the Elapidae family, using two neuronal cell lines, undifferentiated PC12 and differentiated mHippoE-18, in response to H_2_O_2_-induced oxidative stress. Cells were pre-treated for 4 h with PF (10, 1, 0.01, and 0.001 μg mL^−1^), and thereafter exposed to H_2_O_2_ (0.5 mmol L^−1^) for 20 h. Then, the oxidative stress markers and label-free differential proteome strategy were analyzed to understand the neuroprotective effects of PF. In PC12 cells, PF showed no neuroprotective effects against oxidative stress. In mHippoE-18 cells, PF at 0.01 and 0.001 μg mL^−1^ increased the viability and metabolism of cells against H_2_O_2_-induced neurotoxicity, reducing reactive oxygen species (ROS) generation. Interestingly, PF also exhibited a substantial reduction in baseline ROS levels compared to the control, indicating that PF could have compounds with antioxidant features. The comparative proteomic profiling identified 53 proteins with differential expression related to antioxidant action, catalysis, molecular function regulators, structural molecule activity, translation regulatory activity, ATP, and binding. The PF + H_2_O_2_ group indicated that protein expression is 6% upregulated, 4% downregulated, and 94% unchanged compared to the H_2_O_2_ group. Three significant proteins upregulated in the PF + H_2_O_2_ group, including elongation factor 2 (P58252), proteasome subunit alpha type (E9Q0X0), and E2 ubiquitin-conjugating enzyme (A0A338P786), suggested that PF-mediated neuroprotection happens through translational regulation and the degradation of defective proteins via the proteasome complex. Additionally, differential protein expression in PF changed the metabolism, protein synthesis, synaptic activity, and intracellular transport, suggesting that PF contains the rich mixture of bioactive peptides of interest pharmacologically. Overall, this study offers new opportunities for evaluating whether PF’s neuroprotective features in specific neuronal cells are maintained and to investigate neurodegenerative disease drug development processes.

## 1. Introduction

Snake venom is a rich mixture of enzymatic and non-enzymatic compounds, including bioactive peptides [1,2,3]. Understanding the characteristics and variations in snake venom has led to the discovery of peptides with therapeutic properties of interest for the pharmaceutical industry [4] and the development of some drugs approved for commercialization based on peptides present in snake venom [4]. Previous studies have shown promising results in the bioprospecting of neuroprotective compounds in the low molecular weight fraction and in peptides from the venom of the snakes *Bothrops jararaca* (*B. jararaca*) and *Bothrops atrox* (*B. atrox*), both of family Viperidae, in different models for the study of neurodegenerative diseases [5,6,7,8,9,10,11]. Although the venom of snakes of the family Elapidae contains a wide variety of components with properties of pharmaceutical interest [12], their study for the bioprospecting of neuroprotective compounds is still little explored.

Neurodegenerative diseases (NDs) are characterized by the progressive loss of neurons, usually caused by metabolic or toxic disorders, and can lead to death [13,14]. Neurodegeneration is a complex process that shares many fundamental pathways associated with neuronal dysfunction, including oxidative stress, programmed cell death, neuroinflammation, excitotoxicity, mitochondrial dysfunction, lipid peroxidation, and protein misfolding [13,14,15,16]. Currently, some available therapies aim to improve the symptoms caused by NDs; however, these treatments do not address the underlying neurodegeneration associated with these conditions and often come with significant side effects [14,17]. Thus, given the current therapies available for the treatment of NDs, new searches for effective therapies for these diseases have been conducted based on possible pathways with neuroprotective potential [18].

Oxidative stress is considered a metabolic disorder characterized by an imbalance between the production of free radicals and the defenses of the antioxidant system to neutralize them [19,20,21]. If unchecked and not removed by antioxidant defenses, reactive oxygen species (ROS) cause cellular damage, including protein oxidation, DNA and RNA damage, and lipid peroxidation [22,23,24]. Oxidative stress is described as the basis of many NDs, and the use of in vitro models that mimic oxidative stress to understand the pathogenesis of NDs and to search for treatments for them has been well described [25], including the use of hydrogen peroxide (H_2_O_2_). The main cell lines used to study NDs are SH-SY5Y, SK-N-MC, and PC12 [26].

Snakes of the genus *Naja* spp. belong to the family Elapidae [27]. The snake *Naja mandalayensis* (*N. mandalayensis*) is endemic to the central region of Myanmar and is found in arid areas [28]. The proteomics of the crude venom of *N. mandalayensis* was described in 2021 by Beraldo and collaborators, which revealed a complex mixture of proteins [29]. Among the proteins identified in the venom of *N. mandalayensis*, some already known from snake venoms were identified, such as three-finger toxins (3FTx), phospholipases A2 (PLA2s), snake venom metalloproteinases (SVMPs), L-amino acid oxidases (LAAOs), cysteine-rich venom protein (CRISP), venom 5-nucleotidase (V5N), and venom nerve growth factor (VNGF) [29], with most of the proteins found being the 3FTx (cytotoxins and neurotoxins) [29]. Abdullah and collaborators recently reported the neuroprotective effects mediated by phospholipase A2 (A2-EPTX-NSm1a), isolated from the venom of the snake *Naja sumatrana*, in neuronal cells of the SH-SY5Y type against oxidative stress induced by H_2_O_2_ [30]. PLA2 belongs to the family of proteins with enzymatic activities and are well described as components of the venom of Viperidae and Elapidae snakes [2,4]. Although they have a higher molecular mass than the peptides of interest in the present study (<10 kDa), these results, as well as the variation in elapid venom, show that the study of neuroprotective compounds in the venom of another snake of this family may be promising. Thus, the identification of neuroprotective pharmacological properties of interest found in the venom of snakes from the families Viperidae and Elapidae suggests that other compounds may be found in the venom of other snakes. Therefore, the current study aimed to identify novel neuroprotective compounds in the peptide fraction (PF) of *N. mandalayensis*, a snake from the Elapidae family, against oxidative stress using two cell lines: undifferentiated PC12 cells, derived from rat adrenal medullary pheochromocytoma, which can secrete catecholamines such as norepinephrine and dopamine and are commonly utilized for oxidative stress investigation [31,32,33,34]; and differentiated mHippoE-18 cells, derived from mouse embryonic hippocampus, which exhibit neuronal-like morphological and functional properties along with neuronal markers [35], making them an effective model for investigating neurodegenerative diseases and easier to maintain in culture than primary hippocampal cultures [35].

## 2. Materials and Methods

### 2.1. Reagents and Cell Lines

The chemicals used in this research were obtained from Sigma–Aldrich© (St. Louis, MO, USA) or comparable sources with above 98% analytical purity. The solutions were prepared using deionized water filtered through a 22 μM pore membrane and resistivity above 18.2 Ω (Merck Millipore; Burlington, MA, USA). Two cell lines were employed in the present study: a neuronal PC12 cell derived from a transplantable rat pheochromocytoma (CRL-1721™ from the American Type Culture Collection—ATCC, Manassas, VA, USA); and, hippocampal mHippoE-18 cells derived from a primary culture of embryonic mouse hippocampal cells (CLU199, CELLutions Biosystems, Toronto, ON, Canada).

### 2.2. Crude Venom and Obtaining PF

The crude venom of the *N. mandalayensis* snake was obtained from individuals in captivity [29] and was provided by the Butantan Institute through a partnership with the Ministry of Health of Myanmar. The PF was prepared by Dr. Emidio Beraldo Neto, researcher of the Butantan Institute, using a molecular weight cut-off 10 kDa filter (Amicon Ultracel; Merck KGaA, Darmstadt, Germany) according to the manufacturer’s specifications. The fraction obtained was lyophilized and stored at −20 °C until use.

### 2.3. PF Characterization by Mass Spectrometry

PF was analyzed by mass spectrometry on an ESI-IT-TOF coupled to a UFLC (20A Prominence; Shimadzu Corporation, Kyoto, Japan). The sample was injected into a C18 column (Kinetex C18, 5 μm; 50 × 2.1 mm; Phenomenex, Torrance, CA, USA) in a binary solvent system: solvent A containing water, acid (999:1); and solvent B containing ACN, water, and acid (900:99:1). The elution gradient used was 0–100% of solvent B for 20 min in a constant flow of 0.2 mL·min^−1^ after initial isocratic elution for 5 min. The interface was kept at 4.5 kV and 200 °C. Detectors operated at 1.95 kV. MS spectra were acquired in positive mode in the 300–1900 *m*·*z*^−1^ range.

### 2.4. Culture and Maintenance

PC12 and mHippoE-18 cells were regularly grown in low-glucose (D10) and high-medium (DH10) DMEM (Dulbecco’s Modified Eagle Medium; Thermo Fisher Scientific Inc., Waltham, MA, USA), respectively, with a 10% fetal bovine serum (FBS) supplement (Thermo Fisher Scientific Inc., MA, USA), inactivated at 56 °C in a water bath for 30 min. All the media were also supplemented with 1% (v·v^−1^) of 10,000 U·mL^−1^ penicillin, 10 mg·mL^−1^ streptomycin, and 25 µg·mL^−1^ amphotericin B solutions (Sigma–Aldrich, St. Louis, MO, USA). The culture was kept at 37 °C in a humidified environment that contained 5% CO_2_ and 95% air (Forma™ Series 3 Water Jacketed CO_2_ Incubator; Thermo Scientific Inc., MA, USA). The culture medium was replaced every 2–3 days, and, at 80% confluence, cells were passaged using trypsin–EDTA solution [0.05% (m·v^−1^) trypsin and 0.02% (m·v^−1^) EDTA] on PC12 cells and Versene solution (140 mmol·L^−1^ NaCl, 2.7 mmol·L^−1^ KCl, 10 mmol·L^−1^ Na_2_HPO_4_, 1.8 mmol·L^−1^ KH_2_PO_4_, and 0.5 mmol·L^−1^ EDTA) on mHippoE-18 cells.

### 2.5. Cytotoxic Effects of PF

The assessment was conducted in PC12 and mHippoE-18 cells through a period of 24 h of treatment. Cells were cultured into 96-well plates (Nest Biotechnology, Rahway, NJ, USA), with a cell density of 5 × 10^3^ for PC12 cells and 2.5 × 10^3^ for mHippoE-18 cells per well in 100 μL of D10 or DH10 for 24 h, respectively. Subsequently, cells were treated with PF at 10, 1, and 0.01 μg·mL^−1^, according to previous studies [8,11]. Cell viability was assessed using two methods: cell integrity evaluated with crystal violet dye [36] and metabolic activity by the conversion of resazurin to resorufin [37]. All experiments included untreated cells (negative control) and cells exposed to acrylamide (100 mmol·L^−1^; positive control). Data were shown as box-and-whisker plots of three independent experiments in sextuplicate and represent the percentage of cell integrity or metabolic activity in relation to the control.

### 2.6. Cell Integrity

Following the treatments, the medium was removed, and 50 μL of 0.5% violet crystal solution was introduced to each well. The plate was homogenized for 20 min (20 oscillations per min) (IKA KS 260 basic; IKA-Werke GmbH & Co. KG, Staufen im Breisgau, Germany) at room temperature. Thereafter, the violet crystal solution was removed and the plate was subjected to two washes with deionized water. The plate was completely dried; then, 200 μL of methanol was added. The absorbance of the violet samples was measured at 570 nm using a BioTek Epoch microplate reader (Santa Clara, CA, USA).

### 2.7. Metabolic Activity

Cells were exposed to different conditions in a D10 or DH10 medium containing resazurin at 40 μM. After 24 h of incubation, the fluorescence reading of the resazurin reduction in resorufin was performed at these times in the microplate reader (BioTek Synergy HT Multi Mode Microplate Reader, Santa Clara, CA, USA), with 530 nm excitation and 590 nm emission.

### 2.8. Neuroprotection Against Oxidative Stress in PC12 and mHippoE-18 Cells

#### 2.8.1. H_2_O_2_-Induced Oxidative Stress Effects

Initially, the H_2_O_2_-induced oxidative stress effects were evaluated in both cell types. PC12 and mHippoE-18 cells (5 × 10^3^ and 2.5 × 10^3^ in 100 μL per well, respectively) were plated in 96-well plates (Nest Biotechnology, Rahway, NJ, USA) and incubated for 24 h. After, the D10 or DH10 medium was replaced and the cells were incubated at 37 °C for 4 h. Then, the medium was removed, and a new medium was added containing different concentrations of H_2_O_2_ (2, 1, 0.5, and 0.3 mmol·L^−1^); the cells were incubated again for 20 h at 37 °C with 5% CO_2_. Untreated cells were included as a control group. Cell viability measured the H_2_O_2_-induced oxidative stress effects in both cells using the crystal violet dye method previously described [36]. Subsequently, the resazurin method [37] and 2′,7′-dichlorodihydrofluorescein diacetate (H_2_DCFDA; Sigma–Aldrich, St. Louis, MO, USA) staining [9,38] were employed to assess the impact of oxidative stress on the metabolic activity and ROS production of cells at a concentration of 0.5 mmol·L^−1^ H_2_O_2_. Data were shown as box-and-whisker plots of three independent experiments in sextuplicate and represent the percentage in relation to the control.

#### 2.8.2. Neuroprotection Assay

In the neuroprotection model, cells were pre-treated for 4 h with cell type-specific culture media containing PF at doses of 10, 1, and 0.01 μg·mL^−1^, according to previous studies [8,11]. After, the culture media was discarded and replaced with a fresh medium containing identical doses of PF and H_2_O_2_ (0.5 mmol·L^−1^) (PF + H_2_O_2_ group), followed by an incubation period of 20 h. Cells that were treated for 4 h only with culture medium and subsequently maintained in that medium (control group) or incubated exclusively with H_2_O_2_ (H_2_O_2_ group) for 20 h were also a part of the experiments. The evaluation of neuroprotective effects was conducted through the assessment of cell integrity, metabolism, and the ROS production. Additional tests were conducted in mHippoE-18 cells, utilizing PF concentrations of 0.001 μg·mL^−1^. Data were shown as the box-and-whisker plots of three independent experiments in sextuplicate and represent the percentage in relation to the control.

#### 2.8.3. ROS Quantification

Briefly, 50 μL of culture medium from the experimental groups were distributed into a 96-well black plate (SPL Life Sciences, Pocheon-si, Republic of Korea), followed by the addition of 145 μL of PBS and 5 μL of H_2_DCF-DA at a concentration of 1 mmol·L^−1^ (Sigma–Aldrich^®^, St. Louis, MO, USA). The plate was incubated at 37 °C for 1.5 h. The fluorescence was subsequently assessed using a microplate reader (BioTek Synergy HT Multi Mode Microplate Reader, Santa Clara, CA, USA), employing 480 nm excitation and 530 nm emission wavelengths. The data were presented as percentages relative to the control, displayed in box-and-whisker plots.

#### 2.8.4. Label-Free Quantitative Mass Spectrometry Analysis

**Figure 1:**
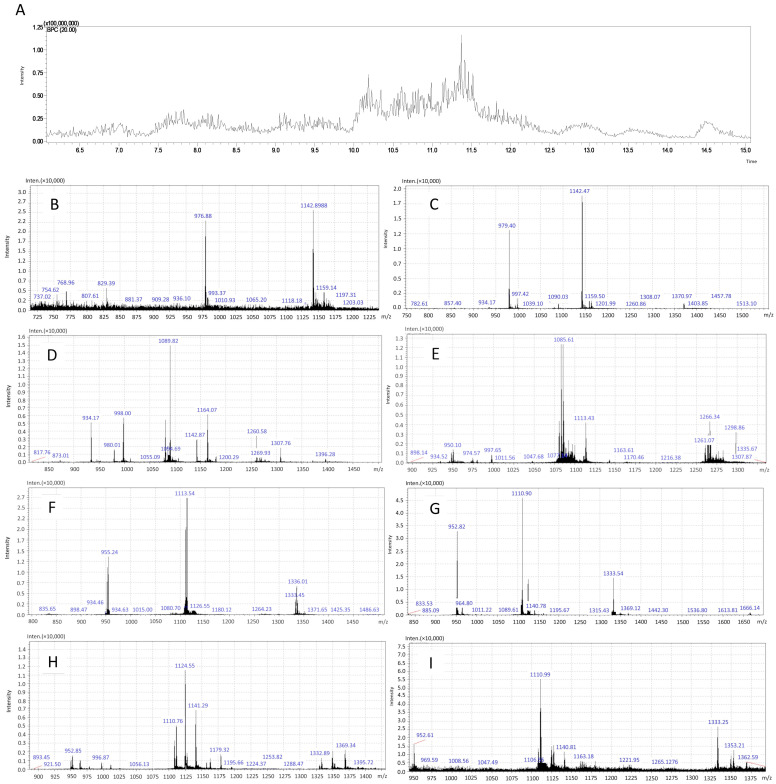
Characterization of PF from *Naja mandalayensis* venom by mass spectrometry. A base peak chromatogram of the total time analyzed and (**A**) a zoomed-in image of the retention time of analyzed peaks from a fraction of *Naja mandalayensis* venom after molecular filtration with a 10 kDa cutoff. The molecular mass analysis confirming the efficiency of the peptide fraction obtention from crude venom. The result displays an intensity analysis conducted by the m/z ratio with the following retention times: (**B**) 6.077 → 7.368 Scan: 730 → 885; (**C**) 7.352 → 7.993 Scan: 883 → 960; (**D**) 7.968 → 8.985 Scan: 957 → 1079; (**E**) 8.960 → 9.993 Scan: 1076 → 1200; (**F**) 9.968 → 11.002 Scan: 1197 → 1321 (**G**) 11.002 → 12.010 Scan: 1321 → 1442; (**H**) 11.985 → 13.010 Scan: 1439 → 1562; (**I**) 13.002 → 15.060 Scan: 1561 → 1808.

The mHippoE-18 cells were seeded at 3.2 × 10^4^ per well in 12-well plates (Nest Biotechnology, Rahway, NJ, USA) and incubated for 24 h. Subsequently, different cell groups (control, H_2_0_2_, PF, PF + H_2_0_2_; 4 wells per group) were either untreated or subjected to treatment following previous experimental design (Figure 1) using 0.001 μg·mL^−1^ PF. Subsequently, the cells were washed with PBS twice, and total protein was extracted using methanol. Protein digestion was carried out directly in solution using cell culture-derived samples. Peptide cleanup was performed employing C18 ZipTips (Merck KGaA, Darmstadt, Germany) to remove contaminants prior to mass spectrometry analysis. An aliquot (1 µL) of the tryptic digest was analyzed via nano-electrospray ionization liquid chromatography tandem mass spectrometry (nano-ESI-LC-MS/MS) on a Dionex Ultimate 3000 RSLCnano system (Thermo Fisher Scientific, Waltham, MA, USA), interfaced with an Impact II mass spectrometer (Bruker Daltonics, Bremen, Germany). Peptide separation was initiated using a nano-trap Acclaim PepMap column (Dionex-C18, 100 Å, 75 μm × 2 cm) in 2% solvent A2 (0.1% formic acid) for 2 min at a flow rate of 5 µL·min^−1^. Peptide elution followed a linear gradient from 5% to 40% solvent B2 (0.1% formic acid in acetonitrile) over 120 min under a flow rate of 350 µL·min^−1^. Data acquisition was performed in positive ion mode, with MS and MS/MS scans being collected at 2 Hz over a mass range of 50–2000 m/z. Collision-induced dissociation (CID) was employed with energy ramping from 7 to 70 eV. Proteomic data processing was conducted using PEAKS Studio 8.5 (Bioinformatics Solution Inc., Waterloo, ON, Canada), with protein identification being performed against the UniProt Mus musculus reference proteome (UP000000589). Quantitative label-free proteomics were executed via the PEAKS Q module, incorporating chromatographic alignment, peptide identification, and relative quantification using the Peaks DB search algorithm. Peptide abundance variations were assessed by comparing peak areas extracted from ion chromatograms. The analysis parameters included a precursor ion mass tolerance of 15 ppm with peptide intensities normalized by total ion current (TIC). Ratio estimations between experimental conditions were filtered according to the software’s automated criteria [39].

### 2.9. Statistical Analyses

The data were tested for unequal variance and normality. Statistical analyses were conducted using a one-way analysis of variance (ANOVA) to compare differences between groups, followed by Tukey’s post hoc test for multiple comparisons or for assessing multiple treatments against a single control. Statistical analysis with *t*-test were performed for the oxidative stress model trials. A significance threshold of *p* ≤ 0.05 was applied. All analyses were performed using GraphPad Prism 6.0 (GraphPad Software, Inc., La Jolla, CA, USA) and values were presented as box-and-whisker plots from three independent experiments in sextuplicate. Proteomic analyses were carried out using the PEAKSQ software (https://www.bioinfor.com/quantification/; accessed on 15 December 2024), utilizing its internal statistical models to identify proteins with altered expression across the experimental conditions.

## 3. Results

### 3.1. PF Analysis by Mass Spectrometry

PF was obtained by molecular cutting of the crude venom in Amicon Ultracel filter (10 kDa) and characterized by mass spectrometry using the LC-ESI-IT-MS/MS method. The chromatographic profile of PF indicated that the retention time of most compounds occurred from 6 to 15 min (Figure 1A). The molecular mass analysis of PF compounds with retention times between 6 and 15 min indicated that the present components have a molecular mass of less than 10 kDa when analyzing the relations between intensity and mass/charge ratio (m·z^− 1^) (Figure 1B–I).

### 3.2. Toxicological Profile of PF

**Figure 2:**
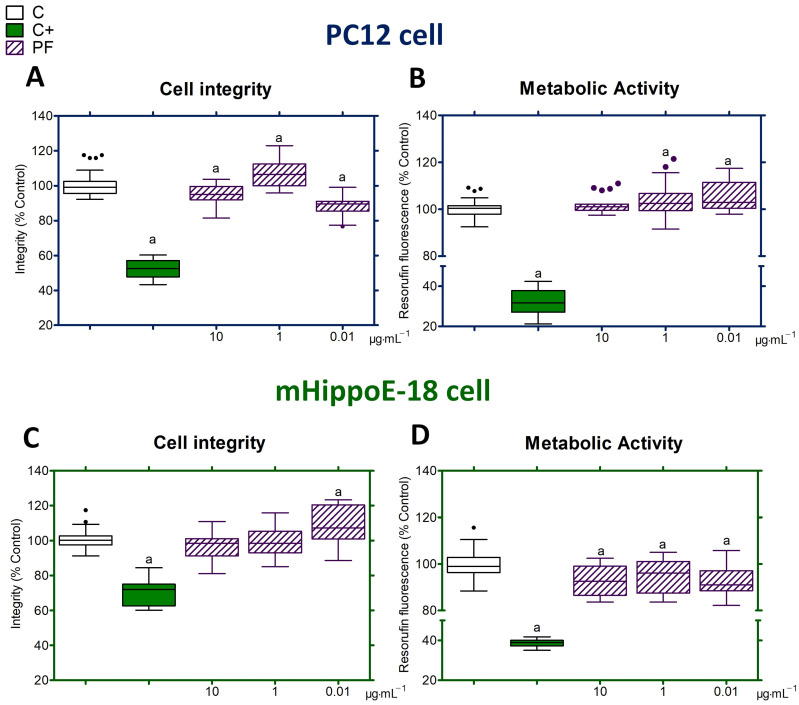
Toxicity of PF in neuronal PC12 cells and hippocampal mHippoE-18 cells. The viability of the cells was assessed by examining their integrity (**A**,**C**) and metabolism (**B**,**D**) after 24 h of PF treatment. (a) Statistical significance was observed with *p ≤* 0.05 compared to the control group. Values that exceed the whiskers were shown as dots. C: untreated cell group. C+: cell group treated with acrylamide. PF: cell group treated with a peptide fraction.

PC12 cells treated with PF for 24 h decreased in cell viability at 10 and 0.01 μg·mL^−1^ but increased at 1 μg·mL^−1^ when compared to the control group (Figure 2A). PF at 1 and 0.01 μg·mL^−1^ enhanced the metabolic activity of PC12 cells; however, this effect was not observed at a concentration of 10 μg·mL^−1^ after 24 h of treatment (Figure 2B). In mHippoE-18 cells, PF increased cell viability only at a concentration of 0.01 μg·mL^−1^, relative to the control group (Figure 2C). The metabolic activity of mHippoE-18 cells treated with PF decreased at all tested concentrations (Figure 2D). Acrylamide (100 mmol·mL^−1^) in both cell types under the same treatment conditions resulted in decreased integrity and metabolism compared to the control (Figure 2A–D).

### 3.3. Oxidative Stress Model in PC12 and mHippoE-18 Cells

**Figure 3:**
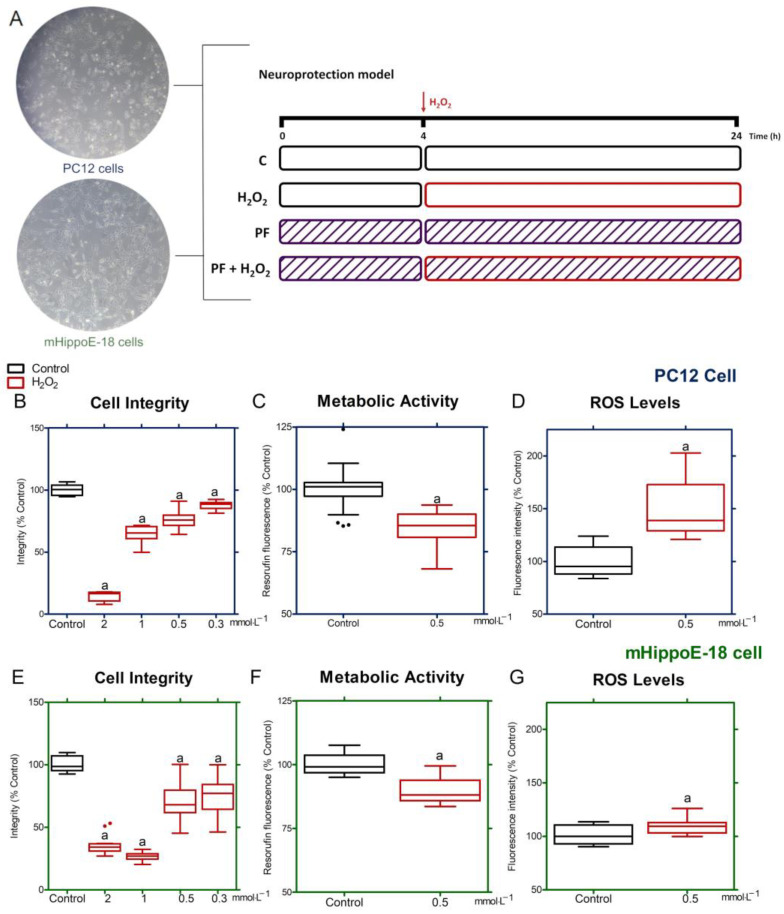
Effects of H_2_O_2_-induced oxidative stress on PC12 (^**▬**^) and mHippoE-18 cells (^**▬**^). Schematic representation of neuroprotection model adopted in PC12 and mHippoE-18 cells, as shown in a typical photomicrograph (magnification: ×80) (**A**). Both cell types were exposed to different concentrations of H_2_O_2_ (2, 1, 0.5, and 0.3 mmol·L^−1^); after 20 h, the cell integrity was evaluated (**B**,**E**). The concentration of 0.5 mmol·L^−1^ was selected for the analysis of cellular metabolism (**C**,**F**) and production of ROS (**D**,**G**) in the cells PC12 and mHippoE-18, respectively. Values that exceed the whiskers were shown as dots. (a) *p ≤* 0.05 for differences in relation to the control group.

Cells were pre-treated for 4 h at 37 °C with PF diluted in D10 or DH10 or only culture medium. After that, the media were replaced by media containing PF or/and H_2_O_2_ and incubated for 20 h more (Figure 3A). PC12 cells exposed to H_2_O_2_ at 2, 1, 0.5 and 0.3 mmol·mL^−1^ for 20 h exhibited a dose-dependent loss of cellular integrity (Figure 3B). The 0.5 mmol·mL^−1^ H_2_O_2_ diminished cell integrity by 23.67 ± 7.04% and cellular metabolism by 15.55 ± 6.65% (Figure 3C) while also elevating ROS generation by 48.7 ± 25.8% relative to the control (Figure 3D). In mHippoE-18 cells, a decrease in integrity was detected corresponding to the tested concentrations of H_2_O_2_ (Figure 3E). In these cells, H_2_O_2_ (mmol·mL^−1^) diminished integrity by 28.89 ± 15.68%, decreased cellular metabolism by 10.24 ± 4.85% (Figure 3F), and elevated ROS generation by 9.5 ± 7.7% (Figure 3G).

### 3.4. PF-Mediated Neuroprotection Against Oxidative Stress in PC12 and mHippoE-18 Cells

**Figure 4:**
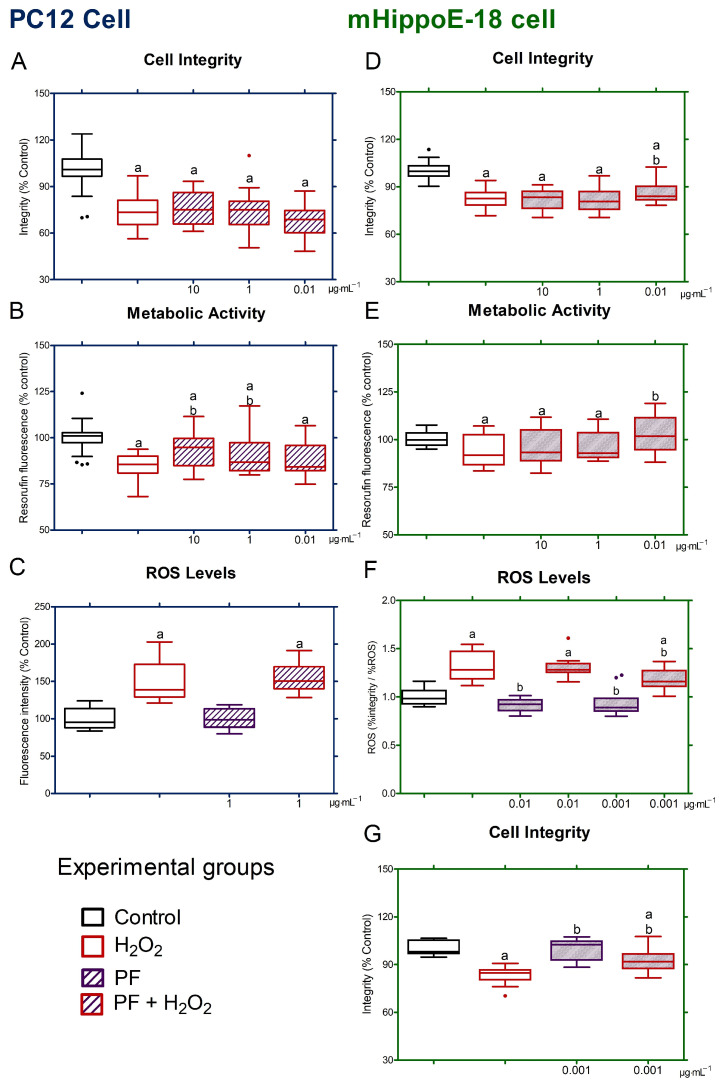
PF-mediated neuroprotection on oxidative stress-induced changes in PC12 and mHippoE-18 cells. PF effects at 10, 1, and 0.01 μg·mL^−1^ against oxidative stress-induced neurotoxicity were evaluated on cell integrity (**A**,**D**), metabolic activity (**B**,**E**)**,** and ROS generation (**C**,**F**) in both cell types. Additional experiments were performed with PF at 0.001 μg·mL^−1^ in mHippoE-18 cells (**G**). Values that exceed the whiskers were shown as dots. (a) *p ≤* 0.05 for differences in relation to control; (b) *p ≤* 0.05 for differences in relation to H_2_O_2_.

The cell integrity of the PF + H_2_O_2_ group had no effect on the damage induced by H_2_O_2_ at all tested concentrations when compared to the results from the H_2_O_2_ group (Figure 4A) in PC12 cells. The PF + H_2_O_2_ group exhibited significantly higher cell metabolism at 10 and 1 μg·mL^−1^ relative to the H_2_O_2_ group (Figure 4B). Despite this, the PF + H_2_O_2_ maintained levels of ROS generation comparable to the H_2_O_2_ group (Figure 4C). Cells in the H_2_O_2_ group exhibited diminished cell integrity and metabolic activity, with elevated ROS levels, in comparison to the control group (Figure 4A–C). In mHippoE-18 cells submitted to oxidative stress (H_2_O_2_ group), the cell integrity and metabolism were reduced compared to the control, but these were restored when the cells were also treated with PF at 0.01 μg·mL^−1^ (PF + H_2_O_2_ group) (Figure 4D,E). Also, cells treated with PF + H_2_O_2 _at 0.01 μg·mL^−1^ increased metabolic activity compared to H_2_O_2 _group (Figure 4E). When ROS generation was measured in cells treated with PF + H_2_O_2_ at various doses, the reduction in ROS levels at 0.001 μg·mL^−1^ was observed in relation to the H_2_O_2_ group (Figure 4F). Furthermore, the PF group produced less ROS than the control group at doses of 0.01 and 0.001 μg·mL^−1^ (Figure 4F). After analyzing the integrity and metabolism of the PF + H_2_O_2_ group, a lower concentration of PF (0.001 μg·mL^−1^) was evaluated for preserving integrity. Cells treated with this dose showed higher integrity than cells treated with H_2_O_2_ (Figure 4G). The relation between ROS production and cell viability in each condition indicated that the H_2_O_2_ group exceeded the control group, whereas the PF + H_2_O_2_ group at 0.001 μg·mL^−1^ was lower than the H_2_O_2_ group (Figure 4F).

### 3.5. Quantitative Proteomics and Network Analysis

**Figure 5:**
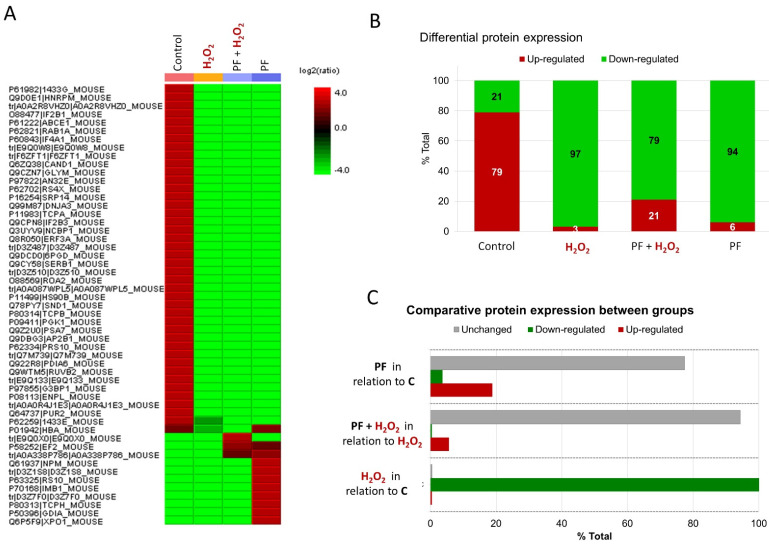
Characterization of differentially expressed proteins of four experimental groups by PEAKS Studio proteomics software (Version X, https://www.bioinfor.com/peaks-studio/ accessed on 15 December 2024). (**A**) Label-free proteomic analysis presented in the heatmap layout, used to calculate the ratio of relative abundances. (**B**) Differential protein expression of total proteins classified as upregulated or downregulated for each experimental group. (**C**) Differential protein expression of total proteins classified as upregulated, downregulated, or unchanged between different groups.

An proteomic label-free analysis was employed to understand the physiological phenomenon regarding cellular repair and the metabolic pathways involved in oxidative stress caused by H_2_O_2_ demonstrated by PF at 0.001 μg·mL^−1^. The analysis identified 53 proteins (Supplementary File S1), and differential protein expression was analyzed through four experimental groups (Control, H_2_O_2_, PF + H_2_O_2_, and PF), as represented in the heatmap (Figure 5A). A gene ontology (GO) analysis generally associated these proteins with signaling pathways for epidermal growth factor and fibroblast growth factor (EGF and FGF), purine and serine glycine biosynthesis, glycolysis, the pentose phosphate pathway, and the ubiquitin proteasome pathway, all of which have been related to Parkinson’s Disease (Supplementary File S2). The molecular function analysis identified activities related to antioxidant action, catalysis, molecular function regulators, structural molecule activity, translation regulatory activity, ATP, and binding.

In the GO molecular function analysis, the control group had an overexpression of approximately 79% of the total proteins identified (Figure 5B), many of which were linked to the binding process and catalytic activity. The H_2_O_2_-induced oxidative stress presented 97% of the proteins that were downregulated, including those in relation to control (Figure 5B,C). PF + H_2_O_2_ group presented 79% downregulated and 21% upregulated 53 proteins (Figure 5B). Also, PF + H_2_O_2_ indicated that 6% are upregulated, 4% are downregulated, and 94% are unchanged protein expression when compared to the H_2_O_2_ group (Figure 5C). Three important upregulated proteins in the PF + H_2_O_2_ group were associated with binding, catalytic activity, and translation regulation. It is interesting that the proteasome subunit alpha type (E9Q0X0) is elevated exclusively in the PF + H_2_O_2_ group, whereas it decreased in the other groups. Additionally, PF + H_2_O_2_ group showed greater levels of key proteins, such as elongation factor 2 (P58252) and the protein synthesis of protein (A0A338P786). The PF group had approximately 21% of proteins in upregulation and 79% in downregulation (Figure 5B) in relation to total protein, which included the molecular function of binding, the regulatory activity of the molecular function, and structural molecular activity. Furthermore, PF had 19% upregulated, 77% downregulated, and 4% are unchanged in relation to control.

## 4. Discussion

The exploration of pharmaceutical compounds for neurodegenerative disease therapy has been highlighted, particularly the biological prospecting of molecules derived from snake venom with therapeutic and neuroprotective properties [1,2,40]. The snake venoms of the Elapidae family have considerable diversity in regard to components of pharmacological relevance, and there is still a lack of research on the bioprospecting of neuroprotective compounds within this group. This study presents, for the first time, that the PF comprising low molecular mass fraction (<10 kDa) derived from the venom of the *N. mandalayensis* snake exhibits neuroprotective properties against oxidative stress-induced neurotoxicity in differentiated mHippoE-18 cells but not in undifferentiated PC12 cells.

Peptide fraction analyses of snake venom have been well reported in the literature using conventional or modern protein chemistry techniques, including mass spectrometry [6,8,29,41,42]. Martins and colleagues studied the PF derived from the venom of the *B. atrox* snake (<14 kDa) using size exclusion chromatography (SEC) and subsequent sodium dodecyl sulfate–polyacrylamide gel electrophoresis (SDS-PAGE) [6]. Querobino and colleagues characterized the PF derived from the venom of the *B. jararaca* snake (<10 kDa) via vibrational spectroscopy methods, SDS-PAGE, gelatinolytic activity assays, and mass spectrometry [8]. In the present study, we employed the mass spectrometry (LC-ESI-IT-TOF-MS) for the PF characterization of *N. mandalayensis* venom, demonstrating high sensitivity for molecular identification. Our results indicated that the obtained PF contains no proteins exceeding 10 kDa.

The cytotoxic effects of PF were evaluated on the integrity and metabolism of neuronal cells of type PC12 and mHippoE-18 after 24 h of treatment at different concentrations. The concentrations of 10 and 0.01 μg·mL^−1^ exhibited cytotoxic effects, but 1 μg·mL^−1^ enhanced the integrity and metabolism of PC12 cells. In mHippoE-18 cells, PF at 0.01 μg·mL^−1^ enhanced the integrity and decreased cellular metabolism, whereas other concentrations exhibited no evidence of cytotoxicity on integrity, but there are compounds in the PF that change the metabolism of these cells. The PF effects on cellular integrity and metabolism in PC12 and mHippoE-18 cells appear to correlate with the evaluated concentration, suggesting that the concentration of specific components within the PF composition is important to their distinct effects. PC12 cells are undifferentiated but have dopaminergic neurons characteristics [31,32,33,43]. The mHippoE-18 cells are differentiated, immortalized cells originating from primary cultures of mouse hippocampal cells [38]. Consequently, different differentiation conditions and phenotypes might be correlated with the different effects of PF on cell viability, suggesting further investigation.

PC12 and mHippoE-18 cells have been employed in neuroprotection experiments against oxidative stress-induced neurotoxicity [11,42,44]. H_2_O_2_ is commonly used as a model for oxidative stress conditions and to investigate the protective effects of pharmacologically relevant compounds [19,22,45,46,47,48]. ROS are compounds that have only one electron in their last valence layer, which makes them highly reactive [49]. Neuronal cultures exposed to H_2_O_2_ cause an imbalance in energy metabolism and can lead to the occurrence of deleterious effects of hydroxyl radicals and membrane proteins [50,51], increasing lipid peroxidation [8]. In our study, PC12 and mHippoE-18 cells exposed to H_2_O_2_ reduced cell integrity in a concentration-dependent manner, according to reports in the literature [7,11,52]. At a concentration of 0.5 mmol·L^−1^, H_2_O_2_ diminished metabolic activity and increased ROS production in both cell types; however, mHippoE-18 cells demonstrated greater resistance to oxidative stress-induced toxicity than PC12 cells due to mHippoE-18 being differentiated neuronal cells that develop more effective antioxidant and repair mechanisms during maturation [53,54]. Furthermore, the downregulation of H_2_O_2_ group proteins compared to other groups in the label-free analysis indicated that H_2_O_2_-induced damage is associated with the degradation of specific proteins, disruption of mRNA translation (which prevents protein synthesis), and the reactivity of heme group proteins with peroxide, potentially resulting from lipid peroxidation [55].

The neuroprotection mediated by snake venoms compounds against oxidative stress has been reported in the literature [1,2]. In our study, PC12 cells and mHippoE-18 were pre-treated for 4 h with different concentrations of PF and then exposed at H_2_O_2_ (0.5 mmol·L^−1^) in the PF presence and incubated for 20 h more. The PF did not show neuroprotective effects against oxidative stress in PC12 cells, although the cells treated at concentrations of 10 and 1 μg·mL^−1^ increased mitochondrial metabolism in the presence of H_2_O_2_ in relation to cells treated only with H_2_O_2_. Nevertheless, PF at 0.01 μg·mL^−1^ restored the integrity and metabolic activity of mHippoE-18 cells compromised by H_2_O_2_ without changing ROS production due to oxidative stress. However, mHippoE-18 cells treated with only PF at concentrations of 0.01 and 0.001 μg·mL^−1^ exhibited a substantial reduction in baseline ROS levels compared to the control, indicating that PF could have antioxidant features. The number of viable cells present in experimental groups treated with PF (0.01 and 0.001 μg·mL^−1^) in the presence and absence of H_2_O_2_ is a factor that should be included when investigating the generation of ROS against oxidative stress. Indeed, the H_2_O_2_ treatment significantly reduced the viability of cells compared to the PF + H_2_O_2_ group, but there were no significant differences in ROS production. Then, we investigated the relationship between ROS generation and viability in all experimental groups, and we demonstrated that cells treated with PF + H_2_O_2_ exhibit lower levels of ROS than cells exposed just to H_2_O_2_, suggesting that PF possesses components with neuroprotective potential against oxidative stress.

The PF-induced neuroprotection was investigated to understand the possible cellular repair and the metabolic pathways involved in oxidative stress caused by H_2_O_2_ using a proteomic label-free analysis. The proteasome subunit alpha type (E9Q0X0) constitutes a component of the proteasome complex responsible for the degradation of defective proteins [56] which was found upregulated in the PF + H_2_O_2_ group, possibly modulating H_2_O_2_-induced oxidative stress. The PF + H_2_O_2_ group also exhibited significant upregulation of key proteins in addition to the PF group, specifically elongation factor 2 (P58252), facilitating the translocation of tRNAs and mRNAs within the ribosome [57]. Additionally, the E2 ubiquitin-conjugating enzyme (A0A338P786) is associated with the ubiquitination process, which marks proteins for degradation by the proteasome [58,59]. Then, the restoration of protein expression, including proteasome subunit alpha type, elongation factor 2, and proteins involved in protein synthesis, after PF treatment under oxidative stress could be attributed to several pathq2ways. One process is the activation of the nuclear factor erythroid-2 related factor 2 (Nrf2) pathway, which regulates an extensive spectrum of antioxidant enzymes that detoxify and reduce oxidative damage, involved in cellular defense systems [60]. Moreover, oxidative stress could cause problems with protein folding mechanisms due to its effect on redox homeostasis. The endoplasmic reticulum (ER) is essential for protein folding, and increased oxidative stress may impair its function, resulting in an accumulation of misfolded proteins. The interaction between oxidative stress and endoplasmic reticulum stress is crucial for comprehending biological responses and developing treatment alternatives [61]. Furthermore, oxidative stress also affects translation and protein synthesis, which is typically downregulated [62]. The insights offer a framework for understanding how PF compounds could mitigate oxidative stress effects to enhance protein expression, emphasizing the significance of antioxidant pathways, protein folding mechanisms, and translational control in cellular recovery processes.

A proteomic label-free analysis indicated that cells treated alone with PF exhibited that the protein expression was 77% upregulated and 19% downregulated compared to the control. The differential protein expressions indicate that the composition of PF is complex and includes peptides which affect essential neuronal processes, such as metabolism, protein synthesis, synaptic activity, and intracellular transport. The changes in protein expression suggested the PF induces toxic effects along with cellular compensating efforts in response to stress in mHippo-E18 cells. The most relevant downregulated proteins suggest disruptions in essential neurons, while the upregulated proteins indicate adaptive mechanisms to alleviate these damages. Among the downregulated proteins, heat shock protein HSP90-beta (P11499) and endoplasmin (P08113) are critical for protein folding and stability [63], and their loss stimulates vulnerability to cellular stress. T-complex protein 1 subunit beta (P80314) and alpha (P11983) decreases also affect protein folding and cellular stability [64]. Vesicular transport and organelle distribution are affected by Ras-related protein Rab-1A (P62821) and AP-2 complex subunit beta (Q9DBG3) decreases in intracellular trafficking [65,66]. In terms of energy metabolism, the downregulation of fructose-bisphosphate aldolase A (D3Z510) and 6-phosphogluconate dehydrogenase (Q9DCD0) reduces the consumption of energy [67], whereas the decreased expression of cell signaling proteins, including 14-3-3 gamma (P61982) and epsilon (P62259), reduces cytoskeletal integrity and signal transduction [68]. As a compensatory attempt, the upregulation of proteins such as Elongation Factor 2 (P58252) and ribosomal proteins, e.g., small ribosomal subunit protein uS7 (D3Z1S8) and small ribosomal subunit protein eS10 (P63325)], suggests an effort to maintain protein translation. The increased expression of importin beta-1 (P70168) and exportin-1 (Q6P5F9) reflects a reorganization in nuclear-cytoplasmic transport [69], while the elevation of L-lactate dehydrogenase (D3Z7F0) suggests adaptation to metabolic stress through anaerobic metabolism [70].

Although this work has shown that oxidative stress can be influenced by PF using in vitro protocols, there are limitations, particularly concerning PF’s permeability across the blood–brain barrier, the lack of peptide characterization, and the absence of techniques for analyzing proteins and their interactions. In vivo studies could provide additional insights. Future considerations indicate that mHippoE-18 cells, as a hippocampal cell line, may enhance preclinical investigations in mice to explore the dose–effect relationship in memory and motor assessments following different delivery methods. The neuroprotective potential of PF could primarily be investigated in the hippocampus, prefrontal cortex, and basal ganglia through proteomic and in situ analyses to clarify protein interactions and critical signaling pathways. This would aid in verifying PF’s influence on oxidative stress, neuroinflammation, and proteostasis.

## 5. Conclusions

In summary, we reported for the first time that the peptide fraction obtained from *N. mandalayensis* snake venom exhibited neuroprotective properties against oxidative stress-induced toxicity in differentiated neuronal mHippoE-18 cells derived from the hippocampus. PF-mediated neuroprotection was characterized by a reduction in oxidative stress markers and the overexpression of important proteins involved in the degradation of defective proteins, including proteasome subunit alpha type and E2 ubiquitin-conjugating enzyme. However, a limitation of this study is that the proposed metabolic pathways were not validated through complementary techniques. Additionally, the PF contains a rich mixture of bioactive peptides which act on essential neuronal processes, such as metabolism, protein synthesis, synaptic activity, and intracellular transport; the isolation and identification of specific active peptides within the PF also limits the understanding of the precise components responsible for the observed effects, representing a key objective for future research. Taken together, these findings indicate that there are previously uncharacterized molecules in the PF that could improve the understanding of the findings obtained, including novel applications of pharmacological interest. Therefore, given everything presented, the next steps of this research include the separation, identification, and characterization of these molecules, which may exert the neuroprotective effects observed in mHippoE-18 cells as well as testing in an in vivo model.

## Data Availability

Raw data of mass spectrometry analysis is available at ProteomeXchange, ID PXD059516. (http://proteomecentral.proteomexchange.org/cgi/GetDataset?ID=PXD059516 accessed on 7 January 2025).

## Supplementary Materials

The following supporting information can be downloaded at: www.mdpi.com/article/10.3390/antiox14030277/s1, File S1: Analysis identified proteins; File S2: Gene ontology (GO) analysis.

## Abbreviations

ACN: acetonitrile; ANOVA: one-way analysis of variance; ATP: adenosine triphosphate; AUC: area under the curve; *B. atrox*: *Bothrops atrox*; *B. jararaca*: *Bothrops jararaca;* CO_2_: Carbon dioxide; CRISP: cysteine-rich venom protein; D10: low-glucose medium; DH10: high-glucose medium; DMEM: Dulbecco’s modified Eagle’s medium; DMSO: dimethyl sulfoxide; DTT: dithiotreitol; EDTA: Ethylenediaminetetraacetic acid; EGF: epidermal growth factor; ER: endoplasmic reticulum; ESI-LC-MS/MS: Liquid Chromatography Electrospray Ionization Tandem Mass Spectrometric; 3FTx: three-finger toxins; FBS: fetal bovine serum; FGF: fibroblast growth factor; GO: gene ontology; H_2_DCF-DA: 2′,7′—dichlorodihydrofluorescein diacetate; H_2_O_2_: Hydrogen peroxide; kDa: Kilodalton; LAAOs: L-amino acid oxidases; LC-ESI-IT-MS/MS: Liquid chromatography-electrospray ionization-ion trap-time of flight tandem mass spectrometry; LC-MS/MS: Liquid Chromatography coupled to Tandem Mass Spectrometry; mHippoE-18: neuronal cell line derived from mouse embryonic hippocampus; mRNA: messenger RNA; MS: Mass Spectrometric; MS/MS: Tandem Mass Spectrometry; NDs: neurodegenerative diseases; *N. mandalayensis*: *Naja mandalayensis*; Nrf2: nuclear factor erythroid-2 related factor 2; PBS—phosphate-buffered saline; PC12: neuronal cell line derived from a transplantable rat pheochromocytoma; PF: peptide fraction; PLA2s: phospholipases A2; ROS: reactive oxygen species; SD: standard deviation; SDS-PAGE: subsequent sodium dodecyl sulfate- polyacrylamide gel electrophoresis; SEC: size exclusion chromatography; SH-SY5Y: neuronal cell line derived from a metastatic bone tumor; SK-N-MC: neuronal cell line derived from Askin tumor; SVMPs: snake venom metalloproteinases; TFA: trifluoroacetic acid; TIC: total ion current; tRNA: transfer RNA; V5N: venom 5-nucleotidase; VNGF: venom nerve growth factor.
